# Underrecognition of migraine‐related stigmatizing attitudes and social burden: Results of the OVERCOME Japan study

**DOI:** 10.1002/brb3.3547

**Published:** 2024-07-25

**Authors:** Hisaka Igarashi, Mika Komori, Kaname Ueda, Anthony J. Zagar, Dena H. Jaffe, Yasuhiko Matsumori, Takao Takeshima, Koichi Hirata

**Affiliations:** ^1^ Fujitsu Clinic Kawasaki Japan; ^2^ Japan Drug Development and Medical Affairs Eli Lilly Japan K.K. Kobe Japan; ^3^ Real World and Access Analytics Eli Lilly and Company Indianapolis Indiana USA; ^4^ Real World Evidence Cerner Enviza, an Oracle Company (formerly Kantar Health) Jerusalem Israel; ^5^ Sendai Headache and Neurology Clinic Sendai Japan; ^6^ Department of Neurology, Headache Center Tominaga Hospital Osaka Japan; ^7^ Department of Neurology Dokkyo Medical University Mibu Japan

**Keywords:** free‐text terms, Japan, mesh terms, migraine disorders, social burden, social stigma

## Abstract

**Introduction:**

Migraine‐related stigma (MiRS) and social burden is increasingly recognized. We assessed perspectives and attitudes toward migraine in people with and without migraine in Japan.

**Methods:**

OVERCOME (Japan) was a cross‐sectional, population‐based web survey of people with and without migraine (July−September 2020). People with migraine were individuals who met the modified International Classification of Headache Disorders criteria or had self‐reported physician‐diagnosed migraine. People without migraine were selected per quota sampling to represent the Japanese adult population. People with migraine reported their experiences on stigma and social burden and answered how frequently they experienced stigma using the MiRS questionnaire. Associations between MiRS and disability and MiRS and interictal burden were examined using the migraine disability assessment and Migraine Interictal Burden Scale‐4. People without migraine reported their experiences and attitudes toward people with migraine by answering an 11‐item attitudinal migraine questionnaire.

**Results:**

A total of 17,071 and 2008 people with and without migraine, respectively, completed the survey. Overall, 11,228 (65.8%) respondents with migraine reported that they have never experienced stigma or burden; however, of the 12,383 employed respondents, 5841 (47.2%) reported that their current employers are not “extremely” or “very” understanding about their conditions. Moreover, ∼30%−40% of respondents “sometimes,” “often,” or “very often” hid their migraine from others. The proportion of respondents who experienced stigma often or very often, as assessed by MiRS, was 16.5%; this increased with the increasing number of monthly migraine headache days. The proportion of respondents with moderate‐to‐severe disability and interictal burden increased with increasing stigma. Among respondents without migraine, the proportion holding a stigmatizing attitude toward those with migraine was low (<15%); ∼80% had never experienced work‐ or family‐related stigma or burden.

**Conclusion:**

MiRS and burden exist but may be hidden and underrecognized in Japan. Disease awareness and education may be important to prevent and reduce stigma and burden.

## Graphical abstract

We conducted a cross‐sectional, population‐based web survey to assess perspectives and attitudes toward migraine in people with and without migraine in Japan. MiRS and social burden appeared low in people with and without migraine; however, people with migraine tended to hide their migraine from others, and people without migraine may have a lack of knowledge about migraine and not recognize the disease. This study suggests the importance of early and improved disease recognition, disease awareness, and education because the stigma and burden of migraine may be hidden and underrecognized in Japan.

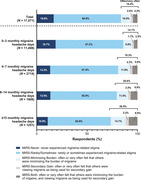

## 1 INTRODUCTION

Migraine is a recurrent, debilitating neurological disease that substantially affects quality of life (Ferrari et al., 2022; Lipton et al., 2003). Headache, the main symptom of migraine, can be frequent, severe, and long‐lasting, limiting or preventing participation in daily activities. Many patients also experience other migraine‐associated symptoms, such as nausea, vomiting, and extreme sensitivity to light and sound. Given that migraine is estimated to affect ≥1 in 10 people worldwide, mainly young adults and middle‐aged women (Safiri et al., 2022; Woldeamanuel & Cowan, 2017), the detrimental effects of migraine are considerable on both a personal and a societal level.

In addition to physical symptoms, many people with migraine experience psychological and emotional burdens, including stigma (Parikh & Young, 2019; R. Shapiro et al., 2022; Young et al., 2013). Stigma is a social construct that attaches negative connotations to the stigmatized person, often resulting in devaluation of the individual, discrimination, and loss of social status and/or employment (Parikh & Young, 2019; R. E. Shapiro, 2020; Young et al., 2013). Stigma can also be internalized, wherein the individual anticipates negative reactions from others and may alter their behavior to avoid stigmatizing situations (Parikh & Young, 2019; R. E. Shapiro, 2020; Young et al., 2013), and is especially related to the person's ability to function at work (Young et al., 2013). Migraine‐associated stigma may result from misunderstanding of the disease by the general public, including viewing migraine as “just a headache” (R. E. Shapiro, 2020). The negative effects of migraine‐associated stigma are increasingly recognized (Seng et al., 2022), and advocacy efforts led by the International Headache Society are underway to increase awareness of headache disorders (Dodick et al., 2020). Furthermore, the application of a biopsychosocial approach, which takes into account sociodemographic and lifestyle factors, including stigma, and the need for close cooperation between policymakers, healthcare professionals, and patients, has been proposed to reduce disease burden and improve disease management (Martelletti et al., 2023; Rosignoli et al., 2022; Vaghi et al., 2023).

The ObserVational survey of the Epidemiology, tReatment, and Care Of MigrainE in Japan (OVERCOME [Japan]) was a cross‐sectional, population‐based web survey designed to examine the epidemiology, medication use, and burden of migraine in Japan (Hirata et al., 2021). In its United States counterpart, OVERCOME (US) (Lipton et al., 2022), nearly one‐third (31.7%) of people with migraine experienced migraine‐related stigma (MiRS) often or very often (R. E. Shapiro et al., 2024). People who experienced greater stigma also experienced more migraine‐associated disability, higher levels of burden between migraines (interictal burden), and lower quality of life. Although the level of stigma was related to the frequency of migraine headaches, even people with the lowest frequency (<4 monthly migraine headache days) had migraine‐associated stigma (25.4%). In this report, we present findings from the OVERCOME (Japan) study related to stigma, including perspectives from people with and without migraine.

## 2 MATERIALS AND METHODS

### 2.1 Study design and participants

OVERCOME (Japan) was a cross‐sectional, population‐based web survey conducted between July and September 2020, which included people with and without migraine who were drawn from a representative of the Japanese population. The study was approved by the Research Institute of Healthcare Data Science Ethical Review Board (ID: RI2020003) and was conducted in accordance with the ethical principles of the Declaration of Helsinki 1964. Informed consent was provided by all survey respondents, and all data in this study were anonymized before analysis.

As previously described (Hirata et al., 2021), participants eligible for this study were adults who resided in Japan and could understand Japanese, had access to the internet, were members of the Kantar Profiles (Lightspeed) online global survey panel or its panel partner (led by Cerner Enviza, formerly Kantar Health), and who could provide electronic informed consent. Targeted sampling to represent the Japanese adult population was applied, using planned demographic sample size quotas for sex and age, which were based on Japanese data retrieved from the US Census Bureau International Database, and for geographic region, based on regional population distribution data from the Statistics Bureau Ministry of Internal Affairs and Communications of Japan (accessed in April 2019). People with migraine were identified as individuals who reported a headache or a migraine attack in the past 12 months and who met the modified International Classification of Headache Disorders, 3rd edition (ICHD‐3) criteria (Headache Classification Committee of the International Headache Society [IHS], 2018), which was assessed by a validated American Migraine Study/American Migraine Prevalence and Prevention Study migraine diagnostic questionnaire (Lipton et al., 2001; Stewart et al., 1992) or had self‐reported physician diagnosis of migraine. People without migraine were defined as individuals without active migraine in the past 12 months and with no self‐reported physician migraine diagnosis, and were identified using quota sampling.

### 2.2 Outcome measures

#### 2.2.1 Stigma and burden of migraine in people with migraine

For people with migraine (migraine group), respondents reported if they had ever experienced any relationship issues due to their migraine. Respondents who were part‐time or full‐time employees were asked how understanding their current employer is about their migraine using a five‐point Likert scale, with responses of “not at all understanding,” “a little understanding,” “somewhat understanding,” “very understanding,” and “extremely understanding,” or “do not remember.” Respondents also reported how often they hide their migraine from others using a five‐point Likert scale, with responses of “never,” “rarely,” “sometimes,” “often,” “very often,” or “not applicable.”

Additionally, MiRS was assessed using the 12‐item MiRS questionnaire (R. E. Shapiro et al., 2024), translated into Japanese (not validated). MiRS items were categorized into two factors, “secondary gain” and “minimizing burden,” and each was scored on a five‐point Likert scale, with responses of “never” (0), “rarely” (1), “sometimes” (2), “often” (3), or “very often” (4). The “secondary gain” factor contained eight items that assessed how frequently the respondents felt that others viewed migraine as being used to obtain or avoid something. The total response scores were categorized as 0 = never, 1−8 = rarely, 9−16 = sometimes, 17−24 = often, and 25−32 = very often. The “minimizing burden” factor contained four items that assessed how frequently the respondents felt that others minimized the burden of their migraine. Categories for the total response score were 0 = never, 1−4 = rarely, 5−8 = sometimes, 9−12 = often, and 13−16 = very often. Based on these categorizations of “secondary gain” and “minimizing burden” factors, respondents were grouped into five overall MiRS groups (R. E. Shapiro et al., 2024): MiRS‐Never (i.e., never for both factors), MiRS‐Rarely/Sometimes (i.e., rarely or sometimes for both factors), MiRS‐Minimizing Burden (i.e., never to sometimes for “secondary gain” and often or very often for “minimizing burden”), MiRS‐Secondary Gain (i.e., often or very often for “secondary gain” and never to sometimes for “minimizing burden”), and MiRS‐Both (i.e., often or very often for both factors).

Using the MiRS questionnaire responses, the association of MiRS on disability and interictal burden of migraine was also assessed. Migraine‐related disability was measured using the Japanese version of the Migraine Disability Assessment (MIDAS), which is a validated five‐item questionnaire reflecting the number of days an individual missed or experienced reduced productivity at work, home, or social events over a 3‐month period (Iigaya et al., 2003; Stewart et al., 2001, 1999). A total MIDAS score is categorized into the following four disability grades: 0−5 = no or little, 6−10 = mild, 11−20 = moderate, and ≥21 = severe. Interictal burden was measured using the Migraine Interictal Burden Scale‐4 (MIBS‐4), translated into Japanese. The MIBS‐4 questionnaire is a validated four‐item instrument measuring migraine‐related burden, including disruption at work and school and diminished family and social life, between migraine attacks (interictal periods) (Buse et al., 2007, 2009). A total MIBS‐4 score ranges from 0 to 12, with the level of interictal burden categorized into a score of 0 = none, 1−2 = mild, 3−4 = moderate, and ≥5 = severe.

#### 2.2.2 Stigmatizing attitudes and burden of migraine in people without migraine

For people without migraine (non‐migraine group), their relationships with a person with migraine were assessed based on the proximity of the relationship (i.e., know no one, a coworker, a friend, and a family member with a migraine) and the number of relationships (know none [0], 1, or ≥2 persons with migraine). These data were combined and categorized into the following five relationship groups: “none,” “coworker only,” “family only,” “friend only,” and “multiple.” Respondents without migraine reported their attitudes toward people with migraine by answering an 11‐item attitudinal questionnaire about migraine, which was developed from qualitative research of focus groups and expert opinion (Table S1) and was translated into Japanese for this study (R. E. Shapiro et al., 2019). The answers were scored on a five‐point Likert scale, with responses of “never,” “rarely,” “sometimes,” “often,” and “very often,” or “do not know”; responses of “do not know,” “never,” or “rarely” were classified as the answer “no,” and responses of “sometimes,” “often,” or “very often” were classified as the answer “yes.” Furthermore, respondents without migraine were also asked about the experiences they have had with migraine at work or with a family member to assess the work‐ and family‐related stigma and burden of migraine.

### 2.3 Statistical analysis

The target sample size was 20,000 individuals for the migraine group and 2000 individuals for the non‐migraine group. All data were described using frequencies and percentages for categorical variables and mean (SD) for continuous variables. For the migraine group, work‐ and relationship‐related stigma and burden were summarized for all respondents and by sex and/or monthly migraine headache days, which were averaged across the past 3 months and categorized as follows: 0−3.99 (0−3), 4.00−7.99 (4−7), 8.00−14.99 (8−14), and ≥15.00 (≥15) days per month. The proportion of respondents in each MiRS group was also summarized for all respondents with migraine and by monthly migraine headache days. Furthermore, the proportion of respondents in each MIDAS and MIBS‐4 category was reported for each MiRS group. For the non‐migraine group, the proportion of respondents who reported “sometimes,” “often,” or “very often” for the 11‐item attitudinal questionnaire was summarized for all respondents without migraine and by the five relationship groups. All statistical analyses were performed using SAS Enterprise Guide 7.15 (SAS Institute, Inc.).

## 3 RESULTS

### 3.1 People with migraine (migraine group)

#### 3.1.1 Demographic and baseline characteristics

A total of 17,071 respondents completed the migraine survey (migraine group). The mean (SD) age was 40.7 (13.0) years, 66.5% were female, 50.1% were married, and 72.5% were part‐time or full‐time employees (Table 1).

**TABLE 1:** Characteristics of survey respondents.

Variable	People with migraine (migraine group) (*N* = 17,071)	People without migraine (non‐migraine group) (*N* = 2008)
Age (years), mean (SD)	40.7 (13.0)^a^, ^b^, ^c^	52.1 (16.7)^a^
Female	11,354 (66.5)^a^, ^b^, ^c^	1048 (52.2)^a^
Married	8555 (50.1)^a^, ^b^	1039 (51.7)^a^
At least some college education	11,891 (69.7)^c^	1349 (67.2)
Employed (full‐time or part‐time)	12,383 (72.5)^a^, ^b^, ^c^	1169 (58.2)^a^
Household income ≥ ¥5,000,000	8131 (47.6)^c^	883 (44.0)
Relationship with the person with migraine		
None	–	1533 (76.3)
Family only	–	300 (14.9)
Friend only	–	95 (4.7)
Coworker only	–	51 (2.5)
Know multiple people with migraine	–	29 (1.4)

*Note*: Data are *n* (%), unless otherwise indicated.

^a^ Previously reported (Hirata et al., 2021).

^b^ Previously reported (Matsumori et al., 2022).

^c^ Previously reported (Takeshima et al., 2022).

#### 3.1.2 Work‐, education‐, and relationship‐related stigma and burden of migraine

Overall, 65.8% (11,228/17,071) of the survey respondents in the migraine group reported that they have never experienced work‐ or relationship‐related stigma or burden with their migraine or severe headache (Table 2). Of the 5843 respondents who reported work‐ or relationship‐related stigma or burden, 52.0% (3040/5843) of respondents reported that they had reduced the number of hours they worked at least once because of their migraine or severe headache. The proportion of respondents who reported work‐ or relationship‐related stigma or burden increased with the increased number of monthly migraine headache days. A numerically greater proportion of male respondents than female respondents generally answered “yes” for each work‐related stigma or burden question (Table 2). The proportion of respondents reporting relationship‐related stigma or burden was similar by sex; however, males answered “yes” more frequently for the questions “had problems with relationship with friends” and “had thought less of yourself as a person.”

**TABLE 2:** Level of internalized work‐, education‐, and relationship‐related stigma and burden by subgroups (migraine group).

“Because of your migraine or severe headache, have you ever…,” yes, *n* (%)	Total (*N* = 17,071)	Monthly migraine headache days				Sex	
		0−3 (*n* = 11,498)	4−7 (*n* = 2714)	8−14 (*n* = 1608)	≥15 (*n* = 1251)	Male (*n* = 5717)	Female (*n* = 11,354)
Work‐ or education‐related stigma							
Lost a job?	251 (1.5)	126 (1.1)	37 (1.4)	29 (1.8)	59 (4.7)	105 (1.8)	146 (1.3)
Had to turn down a job offer or refuse a promotion?	479 (2.8)	252 (2.2)	68 (2.5)	73 (4.5)	86 (6.9)	252 (4.4)	227 (2.0)
Reduced the number of hours you work?	3040 (17.8)	1767 (15.4)	574 (21.1)	389 (24.2)	210 (24.8)	1132 (19.8)	1908 (16.8)
Had to ask an employer to adjust your work schedule or working conditions?	966 (5.7)	530 (4.6)	172 (6.3)	130 (8.1)	134 (10.7)	430 (7.5)	536 (4.7)
Had conflicts with coworkers or supervisors?	507 (3.0)	277 (2.4)	86 (3.2)	67 (4.2)	77 (6.2)	259 (4.5)	248 (2.2)
Not been hired for a job?	869 (5.1)	458 (4.0)	158 (5.8)	122 (7.6)	131 (10.5)	294 (5.1)	575 (5.1)
Had to limit your education or training or change your goals?	1038 (6.1)	572 (5.0)	220 (8.1)	134 (8.3)	112 (9.0)	406 (7.1)	632 (5.6)
Failed a class or had to drop out?	186 (1.1)	107 (0.9)	34 (1.3)	25 (1.6)	20 (1.6)	69 (1.2)	117 (1.0)
Relationship‐related stigma							
Delayed having children, limited the number of children you had, or not had any children?	155 (0.9)	74 (0.6)	32 (1.2)	20 (1.2)	29 (2.3)	60 (1.0)	95 (0.8)
Had problems in relationships with friends?	682 (4.0)	304 (2.6)	146 (5.4)	112 (7.0)	120 (9.6)	288 (5.0)	394 (3.5)
Had problems in relationships with family members?	1177 (6.9)	594 (5.2)	238 (8.8)	162 (10.1)	183 (14.6)	376 (6.6)	801 (7.1)
Had marital or relationship problems?	365 (2.1)	164 (1.4)	67 (2.5)	59 (3.7)	75 (6.0)	127 (2.2)	238 (2.1)
Been accused of being a drug seeker?	409 (2.4)	174 (1.5)	79 (2.9)	72 (4.5)	84 (6.7)	130 (2.3)	279 (2.5)
Thought less of yourself as a person?	607 (3.6)	309 (2.7)	123 (4.5)	77 (4.8)	98 (7.8)	228 (4.0)	379 (3.3)
None of the above	11,228 (65.8)	8033 (69.9)	1657 (61.1)	889 (55.3)	649 (51.9)	3455 (60.4)	7773 (68.5)

Overall, 12,383/17,071 respondents in the migraine group were full‐time or part‐time employees, and of these, 47.2% reported that their current employer is not very understanding (i.e., “not at all,” “a little,” or “somewhat” understanding) when they have a migraine or severe headache; this proportion was 44.8%, 50.9%, 53.1%, and 54.7% in respondents who had 0−3, 4−7, 8−14, and ≥15 monthly migraine headache days, respectively (Table 3). Among the sex subgroup, the proportion of respondents reporting that their current employer is “a little” to “somewhat” understanding was similar between males and females, but those reporting that their current employer is “not at all” understanding was slightly higher in males than females (Table 3).

**TABLE 3:** Level of understanding of current employer toward migraine or severe headache by subgroups (migraine group).

Level of understanding of current employer, *n* (%)	Total (*n* = 12,383)	Monthly migraine headache days				Sex	
		0−3 (*n* = 8462)	4−7 (*n* = 1965)	8−14 (*n* = 1131)	≥15 (*n* = 825)	Male (*n* = 4865)	Female (*n* = 7518)
Extremely understanding	1570 (12.7)	1115 (13.2)	241 (12.3)	118 (10.4)	96 (11.6)	555 (11.4)	1015 (13.5)
Very understanding	2179 (17.6)	1512 (17.9)	344 (17.5)	193 (17.1)	130 (15.8)	864 (17.8)	1315 (17.5)
Somewhat understanding	3756 (30.3)	2530 (29.9)	620 (31.6)	366 (32.4)	240 (29.1)	1472 (30.3)	2284 (30.4)
A little understanding	1312 (10.6)	790 (9.3)	260 (13.2)	145 (12.8)	117 (14.2)	544 (11.2)	768 (10.2)
Not at all understanding	773 (6.2)	469 (5.5)	121 (6.2)	89 (7.9)	94 (11.4)	349 (7.2)	424 (5.6)
Do not remember	2793 (22.6)	2046 (24.2)	379 (19.3)	220 (19.5)	148 (17.9)	1081 (22.2)	1712 (22.8)

The proportion of respondents who reported that they hide their migraine “sometimes,” “often,” or “very often” from coworkers was 38.4%; from a supervisor, boss, or employer was 39.7%; from family was 27.3%; and from friends was 33.5% (Table 4). Among the monthly migraine headache days subgroups, the proportion of respondents hiding their migraine “sometimes,” “often,” or “very often” generally increased with the increasing number of monthly migraine headache days (Table 4). Additionally, a numerically higher proportion of males than females reported that they hide their migraine “sometimes,” “often,” or “very often” (Table 4).

**TABLE 4:** Frequency of hiding migraine by subgroups (migraine group).

“How often do you…,” *n* (%)	Total (*N* = 17,071)	Monthly migraine headache days				Sex	
		0−3 (*n* = 11,498)	4−7 (*n* = 2714)	8−14 (*n* = 1608)	≥15 (*n* = 1251)	Male (*n* = 5717)	Female (*n* = 11,354)
Hide your migraine or severe headache from coworkers							
Never	6146 (36.0)	4343 (37.8)	909 (33.5)	515 (32.0)	379 (30.3)	1948 (34.1)	4198 (37.0)
Rarely	3275 (19.2)	2314 (20.1)	497 (18.3)	273 (17.0)	191 (15.3)	1214 (21.2)	2061 (18.2)
Sometimes	3888 (22.8)	2530 (22.0)	653 (24.1)	398 (24.8)	307 (24.5)	1341 (23.5)	2547 (22.4)
Often	1348 (7.9)	800 (7.0)	241 (8.9)	166 (10.3)	141 (11.3)	494 (8.6)	854 (7.5)
Very often	1314 (7.7)	740 (6.4)	255 (9.4)	154 (9.6)	165 (13.2)	422 (7.4)	892 (7.9)
Not applicable	1100 (6.4)	771 (6.7)	159 (5.9)	102 (6.3)	68 (5.4)	298 (5.2)	802 (7.1)
Hide your migraine or severe headache from a supervisor, boss, or employer?							
Never	6014 (35.2)	4234 (36.8)	905 (33.4)	501 (31.2)	374 (29.9)	1950 (34.1)	4064 (35.8)
Rarely	3052 (17.9)	2161 (18.8)	450 (16.6)	261 (16.2)	180 (14.4)	1126 (19.7)	1926 (17.0)
Sometimes	3604 (21.1)	2351 (20.5)	607 (22.4)	365 (22.7)	281 (22.5)	1243 (21.7)	2361 (20.8)
Often	1578 (9.2)	991 (8.6)	265 (9.8)	182 (11.3)	140 (11.2)	575 (10.1)	1003 (8.8)
Very often	1594 (9.3)	901 (7.8)	315 (11.6)	183 (11.4)	195 (15.6)	500 (8.7)	1094 (9.6)
Not applicable	1229 (7.2)	860 (7.5)	172 (6.3)	116 (7.2)	81 (6.5)	323 (5.6)	906 (8.0)
Hide your migraine or severe headache from family?							
Never	7626 (44.7)	5270 (45.8)	1223 (45.1)	658 (40.9)	475 (38.0)	2309 (40.4)	5317 (46.8)
Rarely	3898 (22.8)	2664 (23.2)	610 (22.5)	384 (23.9)	240 (19.2)	1364 (23.9)	2534 (22.3)
Sometimes	3034 (17.8)	1931 (16.8)	502 (18.5)	305 (19.0)	296 (23.7)	1111 (19.4)	1923 (16.9)
Often	982 (5.8)	600 (5.2)	155 (5.7)	119 (7.4)	108 (8.6)	389 (6.8)	593 (5.2)
Very often	651 (3.8)	395 (3.4)	103 (3.8)	71 (4.4)	82 (6.6)	254 (4.4)	397 (3.5)
Not applicable	880 (5.2)	638 (5.6)	121 (4.5)	71 (4.4)	50 (4.0)	290 (5.1)	590 (5.2)
Hide your migraine or severe headache from friends?							
Never	6670 (39.1)	4691 (40.8)	1019 (37.6)	546 (34.0)	414 (33.1)	2124 (37.2)	4546 (40.0)
Rarely	3710 (21.7)	2553 (22.2)	588 (21.7)	342 (21.3)	227 (18.2)	1267 (22.2)	2443 (21.5)
Sometimes	3716 (21.8)	2372 (20.6)	627 (23.1)	398 (24.8)	319 (25.5)	1271 (22.2)	2445 (21.5)
Often	1202 (7.0)	718 (6.2)	213 (7.8)	144 (9.0)	127 (10.2)	462 (8.1)	740 (6.5)
Very often	798 (4.7)	450 (3.9)	146 (5.4)	94 (5.9)	108 (8.6)	285 (5.0)	513 (4.5)
Not applicable	975 (5.7)	714 (6.2)	121 (4.5)	84 (5.2)	56 (4.5)	308 (5.4)	667 (5.9)

#### 3.1.3 Stigma by MiRS questionnaire

**FIGURE 1:**
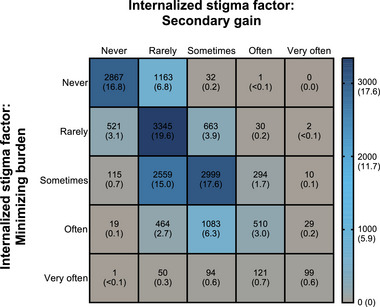
Response distribution matrix for the MiRS questionnaire among respondents with migraine (migraine group). Note: The number of respondents is indicated by the color, which corresponds to the respondent number and percentage shown within the box and the numerical scale. MiRS, migraine‐related stigma.

The frequency of MiRS experienced by respondents in the migraine group was evaluated using the MiRS questionnaire. Overall, 16.8% (2867/17,071) scored “never” for both the “secondary gain” and “minimizing burden” factors for the MiRS questionnaire; only 0.6% (99/17,071) of respondents scored “very often” for both factors (Figure 1). More than a half of the respondents (53.7%; 9175/17,071) scored “sometimes,” “often,” or “very often” for either or both of the “secondary gain” and “minimizing burden” factors of the MiRS questionnaire.

**FIGURE 2:**
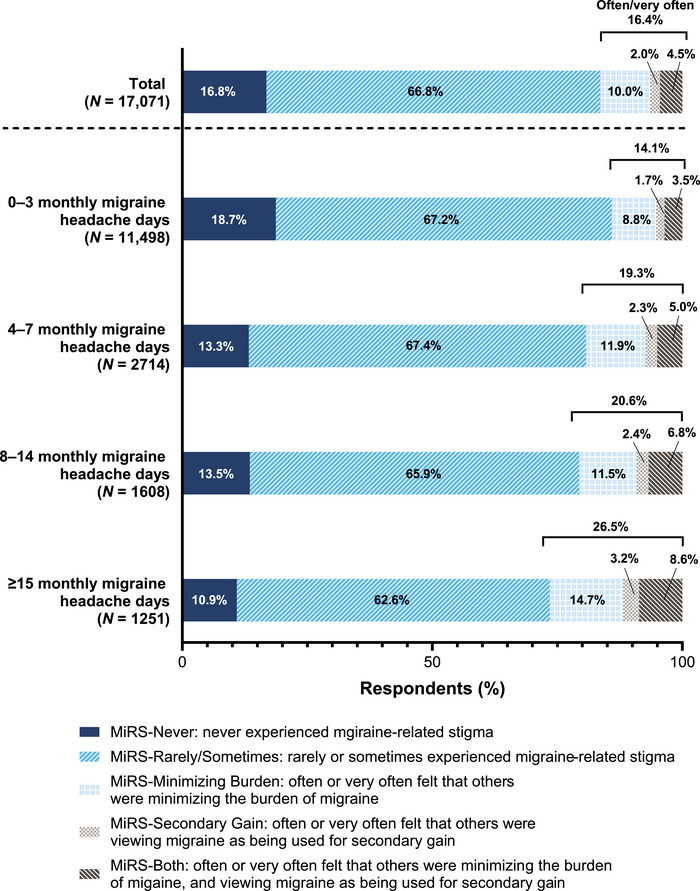
Proportion of respondents with migraine (migraine group) in each MiRS group, stratified by monthly migraine headache days category. MiRS, migraine‐related stigma.

Overall, most respondents with migraine were categorized as MiRS‐Never or MiRS‐Rarely/Sometimes (i.e., reporting that they have never, rarely, or sometimes experienced MiRS) (Figure 2). The proportion of respondents who often or very often felt that others were minimizing their migraine burden or were viewing migraine as being used for secondary gain, or both, was 16.5% (MiRS‐Minimizing Burden: 10.0%; MiRS‐Secondary Gain: 2.0%; and MiRS‐Both: 4.5%). Furthermore, the proportion of respondents who often or very often experienced MiRS related to “minimizing burden” and/or “secondary gain” was almost two times higher in those who had ≥15 monthly migraine headache days (26.5%) than those who had 0−3 monthly migraine headache days (14.0%) (Figure 2).

#### 3.1.4 Relationship between stigma, disability, and interictal burden

**FIGURE 3:**
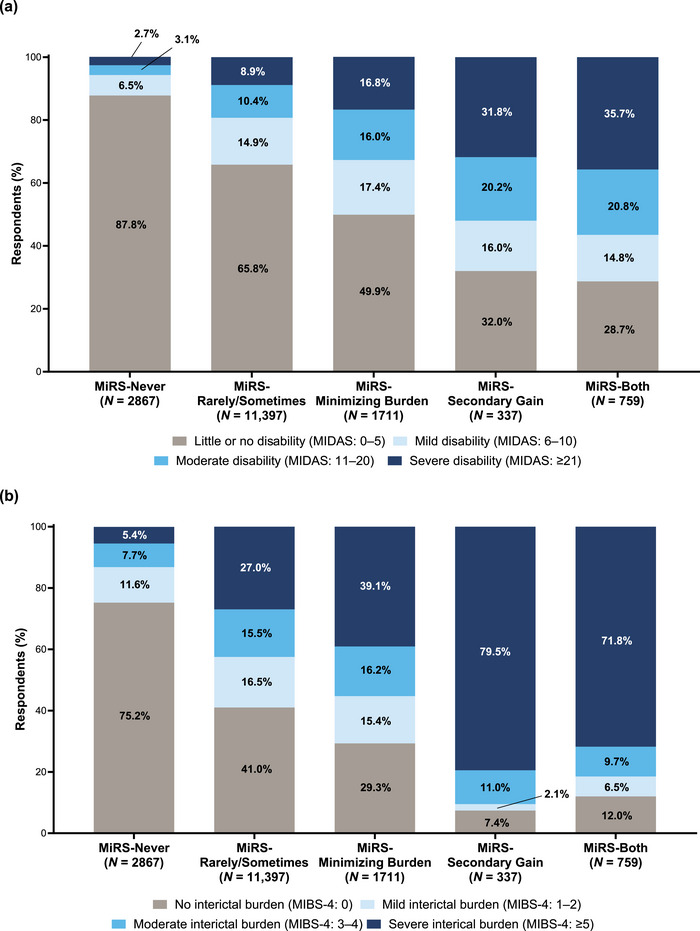
(a) MIDAS category, and (b) MIBS‐4 category by MiRS group. Note: MiRS‐Never: never experienced migraine‐related stigma; MiRS‐Rarely/Sometimes: rarely or sometimes experienced migraine‐related stigma; MiRS‐Minimizing Burden: often or very often felt that others were minimizing the burden of migraine; MiRS‐Secondary Gain: often or very often felt that others were viewing migraine as being used for secondary gain; MiRS‐Both: often or very often felt that others were minimizing the burden of migraine, and viewing migraine as being used for secondary gain. MIBS‐4, Migraine Interictal Burden Scale‐4; MIDAS, migraine disability assessment; MiRS, migraine‐related stigma.

Among respondents in the MiRS‐Never group, most (87.8%) had little or no disability, as assessed by MIDAS, with only 5.8% having moderate‐to‐severe disability (Figure 3a). The proportion of respondents with moderate‐to‐severe disability increased with the increasing frequency of stigma (i.e., MiRS level). Among respondents who had often or very often felt that others were minimizing their migraine burden (MiRS‐Minimizing Burden) or were viewing migraine as being used for secondary gain (MiRS‐Secondary Gain) or both (MiRS‐Both), the proportion of respondents with moderate‐to‐severe disability was 32.8%, 52.0%, and 56.5%, respectively; the proportion with little or no disability ranged from 28.7% to 49.9% in these MiRS groups. The proportion of respondents with moderate or severe interictal burden, as assessed by MIBS‐4, also increased with increasing MiRS level (Figure 3b). Among respondents who never experienced stigma, 75.2% had no interictal burden, with only 7.7% and 5.4% having moderate and severe interictal burden, respectively. Conversely, the proportions of respondents with moderate and severe interictal burden among those who had often or very often felt that others were minimizing their migraine burden (MiRS‐Minimizing Burden) or were viewing migraine as being used for secondary gain (MiRS‐Secondary Gain) or both (MiRS‐Both) were 55.3%, 90.5%, and 81.5%, respectively.

### 3.2 People without migraine (non‐migraine group)

#### 3.2.1 Demographic and baseline characteristics

A total of 2008 respondents without migraine completed the control survey (non‐migraine group) (Hirata et al., 2021). The mean (SD) age of the non‐migraine group was 52.1 (16.7) years, 52.2% were female, 51.7% were married, and 58.2% were part‐time/full‐time employees (Table 1). Most respondents (76.3%) did not know anyone with migraine. The respondent's relationship with the person with migraine was reported as the following: a family member (14.9%; 300/2008), a friend (4.7%; 95/2008), a coworker (2.5%; 51/2008), and multiple relationships (1.4%; 29/2008).

#### 3.2.2 Stigmatizing attitudes toward migraine and burden of working or living with people with migraine

**FIGURE 4:**
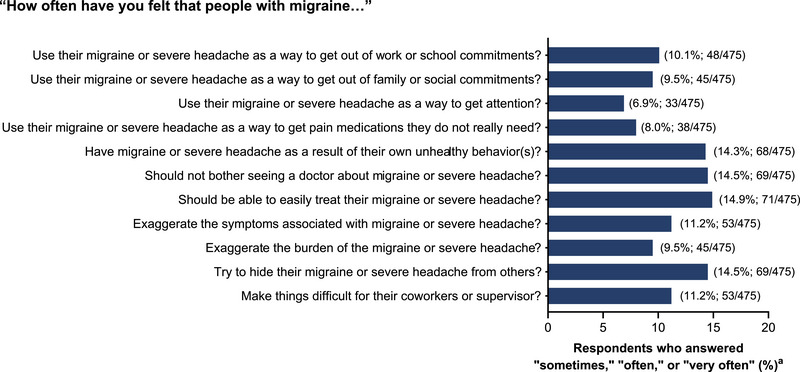
Proportion of respondents without migraine (non‐migraine group) who answered “sometimes,” “often,” or “very often” to the attitudinal questions about migraine. The answers were scored on a five‐point Likert scale, with responses of “never,” “rarely,” “sometimes,” “often,” and “very often,” or “do not know.” ^a^Excluded respondents who did not know anyone with migraine (*n* = 1533).

The attitudes of respondents in the non‐migraine group toward those with migraine were evaluated using the 11 attitudinal questions. Overall, the proportion of respondents (excluding those who did not know anyone with migraine) who “sometimes,” “often,” or “very often” held stigmatizing attitudes toward those with migraine was low (<15%) (Figure 4). The stigmatizing attitudes that had the most respondents answering “sometimes,” “often,” or “very often” were the following: “people with migraine should be able to easily treat their migraine or severe headache” (14.9%), “people with migraine should not bother seeing a doctor about their migraine or severe headache” (14.5%), “people with migraine try to hide their migraine or severe headache from others” (14.5%), and “people with migraine have migraine or severe headache as a result of their own unhealthy behavior(s)” (14.3%). Among the 2008 respondents in the non‐migraine group, the proportion of respondents who “sometimes,” “often,” or “very often” held stigmatizing attitude toward those with migraine was higher in those who knew someone with a migraine than those who knew no one (Figure S1). A greater proportion of respondents who only knew a friend with migraine appeared to have felt that people with migraine “sometimes,” “often,” or “very often” used their migraine as a way to avoid something, to get attention, or to get pain medications that they did not really need, compared with those who had other relationships with people with migraine. Almost 30% of respondents who knew multiple people “sometimes,” “often,” or “very often” felt that people with migraine should be able to easily treat their migraine; this was higher than those who had a relationship only with a coworker, a family member, or a friend with migraine (27.6% vs. 9.8%−17.9%, respectively; Figure S1).

Survey respondents in the non‐migraine group also reported on the experiences they have had with people who have migraines or severe headaches at work (Table 5). Overall, 72.1% (1447/2008) of respondents had never worked with anyone who has migraine or severe headaches. Of the 561 respondents who have worked with someone who has migraine or severe headaches, ∼80% had never experienced any work‐related migraine stigma or burden, including needing to cover work or adjusting their work situation to avoid working with a person with migraine. Of those who have experienced work‐related migraine, the most common work‐related stigma or burden reported was for respondents who “had to work extra hours to cover for a person with migraine” (13.7%).

**TABLE 5:** Stigma and burden of migraine in people without migraine (non‐migraine group).

Stigma/burden Responses, yes, *n* (%)	People without migraine (non‐migraine group) (*N* = 2008)
Work‐related	*n* = 561^a^
Had to cover work for a person with migraine when they lost their job	38 (6.8)
Refused a job offer or promotion to avoid dealing with someone with migraine or severe headaches	24 (4.3)
Worked extra hours to cover for a person with migraine	77 (13.7)
Had to ask an employer to adjust your work schedule or position to avoid working with a person with migraine	27 (4.8)
Had conflicts with coworkers, supervisors, or employees because of a person's migraine or severe headaches	17 (3.0)
None of the above	435 (77.5)
Family‐related	*n* = 321^b^
Had to take time off work to help a person with migraine with their migraine or severe headaches	46 (14.3)
Had to ask an employer to adjust your work schedule to help a person with migraine with their migraine or severe headaches	22 (6.9)
Not been able to take a job or promotion	10 (3.1)
Had conflicts with coworkers or supervisors	7 (2.2)
Had to limit your education or training or change your goals	8 (2.5)
Failed a class or had to drop out	3 (0.9)
Delayed having children, limited the number of children, or not had any children	0 (0.0)
None of the above^c^	253 (78.8)

^a^ Excluded respondents who have never worked with anyone who had migraine or severe headaches (*n* = 1447).

^b^ Respondents with a family member with migraine.

^c^ Exclusive.

A total of 321 respondents who have a family member with migraine reported on the experiences they have had with migraines or severe headaches in the family (Table 5). Almost 80% of respondents had never experienced any stigma or burden related to their family. However, 14.3% of respondents reported that they have had to take time off work, and 6.9% of respondents reported that they have had to ask an employer to adjust their work schedule to help with people's migraine or severe headache. Furthermore, 3.1% of respondents reported that they have not been able to take a job or promotion due to a family member's migraine.

## 4 DISCUSSION

OVERCOME (Japan) is the first population‐based, demographically representative survey to describe and evaluate MiRS and social burden in Japan, examining ∼20,000 and ∼2000 Japanese adults with and without migraine, respectively. Among the survey respondents with migraine, 65.8% of respondents reported that they have never experienced MiRS; almost half (47.2%) of employed respondents reported that their current employers are not very understanding about their conditions; and up to 40% of respondents sometimes to very often hid their migraine from others, including coworkers, family, and friends. Among the survey respondents without migraine, most did not know anyone with migraine (76.3%) or had never worked with people with migraine (72.1%). Moreover, the proportion of respondents without migraine who held stigmatizing attitudes toward people with migraine was low (<15%). Therefore, in this study, MiRS and social burden in people with migraine in Japan existed but did not appear high; however, findings collectively suggest that this may be because people with migraine tended to hide their conditions from others, and people without migraine were not familiar with the disease or its impact on the person or their surroundings, or may not have self‐recognized their own attitudes toward those with migraine.

Migraine is associated with stigma not only from patients’ peers but also from patients’ internalized stigma toward themselves and their disease. Internalized stigma can have a negative impact on an individual's health, especially their ability to work (Perugino et al., 2022; Young et al., 2013). In this study, approximately two‐thirds of respondents with migraine reported that they have not experienced any work‐ or relationship‐related stigma or burden. The OVERCOME (Japan) study previously revealed that, among respondents who met the ICHD‐3 criteria, 43.4% never had a diagnosis for migraine (Hirata et al., 2021; Matsumori et al., 2022), and nearly half of people with migraine have never sought care for their migraine or severe headache (Matsumori et al., 2022). In fact, 36.5% of the people had hesitated to seek care, with the most common reasons being that they felt that their migraine may not be taken seriously and that they did not think their migraine was serious or painful enough. Additionally, another survey study using data from a health insurance association in Japan reported that a majority of patients with migraine endured the symptoms and continued on with their daily activities without resting or seeking care, despite experiencing severe symptoms (Sakai et al., 2022). Together, these results suggest that the stigma and burden of migraine may be affected by patients’ lifestyles, perceptions, and feelings of discomfort with expressing or disclosing their condition to others; people with migraine may be avoiding or trivializing potential stigma by perceiving their migraine as “just a headache,” because they themselves may have a lack of disease awareness and are underestimating their condition. Furthermore, these results may explain why the stigma reported by people with migraine did not appear high in this study.

In this study, people with migraine tended to hide their disease from others. Although further research is required to test this hypothesis, people's actions to hide their condition may be driven by their fear of what others may think of their disease. A cross‐sectional multinational survey in Europe revealed that people with migraine consider greater inclusion in society as the third most important need in terms of quality of life (Vaghi et al., 2023); therefore, hiding their condition may potentially impede patients from being involved in society, ultimately affecting their quality of life. Importantly, results from the current study indicate that, although not apparent or clearly identified, stigma toward migraine exists in Japan and highlight the importance of raising awareness and educating society including patients themselves. Indeed, the biopsychosocial model, supported by recent literature (Perugino et al., 2022; Rosignoli et al., 2022), includes patient education as well as education of society to avoid stigmatization against people with migraine; therefore, this model may lead the future direction of migraine management.

Overall, this study showed that the proportion of respondents experiencing MiRS, as assessed using MiRS questionnaire, often or very often (i.e., categorized as MiRS‐Minimizing Burden, MiRS‐Secondary Gain, and MiRS‐Both) was 16.5%. Consistent with the OVERCOME (US) study (R. E. Shapiro et al., 2024), the proportion increased with the increasing number of migraine headache days. Moreover, the proportion of respondents with severe MIDAS score and MIBS‐4 score was higher in those who experienced MiRS more frequently, suggesting that MiRS is associated with disability and interictal burden, supporting the results from the OVERCOME (US) study that reported that MiRS is associated with more disability, greater interictal burden, and reduced quality of life. However, it should be noted that, among the respondents who often or very often experienced stigma, the proportion of respondents who reported little or no disability ranged from 28.7% to 49.9%; these results indicate that MiRS does exist in a proportion of people with migraine who report little or no disability. Therefore, these results further suggest that efforts to reduce stigma may reduce disability and interictal burden associated with migraine, and conversely, efforts to reduce disability and interictal burden may also help in reducing MiRS.

Overall, the stigma or burden of migraine generally increased with the increasing number of monthly migraine headache days. This may again relate to the fact that people with migraine in Japan are hesitant to seek help and are continuing with their daily activities without resting or seeking support, even if they are suffering from severe symptoms. In this study, work‐related stigma and burden of migraine appeared to be slightly higher in males than females. However, this may be due to the cultural background of a male‐dominated society in Japan; males may possibly be experiencing more stigma and burden because the expectations for them to be strong and successful at work are higher than those of females. Nonetheless, these results do not necessarily suggest that work‐related stigma and burden in females is low; therefore, stigma and burden at work should be considered regardless of sex.

In this study, a small proportion of respondents without migraine (6.9%−14.9%) reported negative attitudes toward people with migraine, which was lower than that reported in the OVERCOME (US) study (23.5%−45.4%) (R. E. Shapiro et al., 2019). Additionally, only approximately 20% of respondents reported that they have experienced any burden associated with having a coworker or family member with migraine. However, in this study, almost 80% of respondents had never experienced any burden of migraine related to work. Again, given that the results from this study suggest that people with migraine tend to hide their migraine from others, a higher prevalence of MiRS and burden may be expected if more people without migraine knew someone with migraine. Additionally, the results observed in this study may be partly explained by the cultural concept in Japan to endure and persevere through difficult situations (“gaman” in Japanese, which is typically translated as “perseverance,” “patient,” “tolerance,” “self‐control,” or “self‐denial”). People with migraine may be enduring their symptoms silently; therefore, many people without migraine may be unaware that a person has a migraine. Moreover, given that the two most common stigmatizing attitudes reported in this study were “people with migraine should be able to easily treat their migraine or severe headache” and “people with migraine should not bother seeing a doctor about their migraine or severe headache,” knowledge about migraine is lacking in Japan. Many people in Japan may be underestimating the effects of migraine on people's lives, ignoring the burden of migraine or viewing migraine as “just a headache,” and considering that people with migraine should be able to endure the symptoms without support. Therefore, results from this study indicate that migraine itself is underrecognized in the general Japanese population; improvement in social awareness and knowledge about migraine may help change perceptions about migraines in society, which could allow people with migraine to openly talk about their conditions (Gross et al., 2023), to ask for support without worrying about stigma from those without migraine, and to allow society to provide appropriate support without stigmatizing the disease.

This was the first large‐scale study to evaluate MiRS and social burden in Japan. A population‐based survey study design enabled the inclusion of a large sample of survey participants that was demographically representative of the general Japanese population. Furthermore, the inclusion of people without migraine enabled the assessment of externalized stigma; internalized stigma in people with migraine was assessed using the validated MiRS questionnaire (although not validated in Japanese). There were several limitations. All surveys were self‐reported; therefore, potential recall bias may exist. Self‐selection may also have led to participant bias; however, this was reduced by using a demographically representative quota sampling method (Hirata et al., 2021). The questionnaire on stigmatizing attitudes toward migraine was derived from qualitative interviews of people without migraine and has not been validated previously.

In conclusion, findings from this study revealed that MiRS and social burden existed but did not appear high in Japan, possibly because people with migraine tend to hide the disease, and thus, migraine is underrecognized in Japanese society. Furthermore, in this study, MiRS was experienced more frequently in people with a greater number of migraine headache days and was associated with a higher disability and interictal burden. Therefore, early recognition of migraine, as well as improved disease awareness and education, not only in people without migraine but in people with migraine, may be important to help prevent people with migraine from experiencing MiRS, disability, and interictal burden.

## AUTHOR CONTRIBUTIONS

**Hisaka Igarashi**: Writing—review and editing. **Mika Komori**: Conceptualization; funding acquisition; supervision; writing—review and editing. **Kaname Ueda**: Conceptualization; funding acquisition; investigation; methodology; project administration; writing—review and editing. **Anthony J. Zagar**: Formal analysis; writing—review and editing. **Dena H. Jaffe**: Data curation; project administration; supervision; writing—review and editing. **Yasuhiko Matsumori**: Writing—review and editing. **Takao Takeshima**: Writing—review and editing. **Koichi Hirata**: Writing—review and editing.

## CONFLICT OF INTEREST STATEMENT

Hisaka Igarashi received personal fees for speaker and consulting services from Amgen K.K., Daiichi Sankyo Company, Limited, Eisai Co., Ltd., Eli Lilly Japan K.K., Otsuka Pharmaceutical Co., Ltd., Sawai Pharmaceutical Co., Ltd., and Takeda Pharmaceutical Company Limited. Mika Komori and Kaname Ueda are employees of Eli Lilly Japan K.K. and own minor shares in Eli Lilly and Company. Anthony J. Zagar is an employee of and owns minor shares in Eli Lilly and Company. Dena H. Jaffe is an employee of Cerner Enviza, an Oracle Company (formerly Kantar Health); Cerner Enviza received research funding for this study from Eli Lilly and Company. Yasuhiko Matsumori received personal fees for consulting services from Amgen Astellas BioPharma K.K., Daiichi Sankyo Company, Limited, Eli Lilly Japan K.K., and Otsuka Pharmaceutical Co., Ltd. Takao Takeshima received personal fees from AbbVie GK, Amgen Astellas BioPharma K.K., Asahi Kasei Pharma Corporation, Bayer Yakuhin, Ltd., Daiichi Sankyo Company, Limited, Eli Lilly Japan K.K., FUJIFILM Toyama Chemical Co., Ltd., Janssen Pharmaceutical K.K., Kowa Company, Ltd., Kyowa Kirin Co., Ltd., Lundbeck Japan K.K., Mitsubishi Tanabe Pharma Corporation, Novartis Pharma K.K., Ono Pharmaceutical Co., Ltd., Otsuka Pharmaceutical Co., Ltd., Pfizer Japan Inc., Sawai Pharmaceutical Co., Ltd., Sumitomo Pharma Co. Ltd., and UCB Japan Co. Ltd. He also received personal fees from Takeda Pharmaceutical Company Limited outside the submitted work. Koichi Hirata received research funding from the Japan Agency for Medical Research and Development and the Japanese Ministry for Health, Labour and Welfare, and personal fees from Amgen Astellas BioPharma K.K., Daiichi Sankyo Company, Limited, Eisai Co., Ltd., Eli Lilly Japan K.K., MSD Co., Ltd., Otsuka Pharmaceutical Co., Ltd., and Pfizer Japan Inc.

### PEER REVIEW

The peer review history for this article is available at https://publons.com/publon/10.1002/brb3.3547.

## Supporting information

TABLE S1 Questions about stigmatizing attitudes toward migraine in the 11‐item attitudinal questionnaire.
FIGURE S1 Proportion of respondents without migraine (non‐migraine group) who answered “sometimes,” “often,” or “very often” to the attitudinal questions about migraine by their relationship to the person with migraine

## Data Availability

The data that support the findings of this study are available from the corresponding author upon reasonable request.

## References

[brb33547-bib-0001] Buse, D. C. , Bigal, M. B. , Rupnow, M. , Reed, M. , Serrano, D. , & Lipton, R. (2007). Development and validation of the Migraine Interictal Burden Scale (MIBS): A self‐administered instrument for measuring the burden of migraine between attacks. Neurology, 68, A89.

[brb33547-bib-0002] Buse, D. C. , Rupnow, M. F. T. , & Lipton, R. B. (2009). Assessing and managing all aspects of migraine: Migraine attacks, migraine‐related functional impairment, common comorbidities, and quality of life. Mayo Clinic Proceedings, 84(5), 422–435. 10.4065/84.5.422 19411439 PMC2676125

[brb33547-bib-0003] Dodick, D. W. , Ashina, M. , Sakai, F. , Grisold, W. , Miyake, H. , Henscheid‐Lorenz, D. , Craven, A. , Ruiz de La Torre, E. , Koh, R. , Reznik, N. , Bance, L. , Leroux, E. , & Edvinsson, L. (2020). Vancouver declaration II on global headache patient advocacy 2019. Cephalalgia, 40(10), 1017–1025. 10.1177/0333102420921162 32345038

[brb33547-bib-0004] Ferrari, M. D. , Goadsby, P. J. , Burstein, R. , Kurth, T. , Ayata, C. , Charles, A. , Ashina, M. , van den Maagdenberg, A. M. J. M. , & Dodick, D. W. (2022). Migraine. Nature Reviews Disease Primers, 8(1), Article 2. 10.1038/s41572-021-00328-4 35027572

[brb33547-bib-0005] Gross, E. , Ruiz de La Torre, E. , & Martelletti, P. (2023). The migraine stigma kaleidoscope view. Neurology and Therapy, 12(3), 703–709. 10.1007/s40120-023-00456-x 36871256 PMC10195931

[brb33547-bib-0006] Headache Classification Committee of the International Headache Society (IHS) . (2018). The International classification of headache disorders, 3rd edition. Cephalalgia, 38(1), 1–211. 10.1177/0333102417738202 29368949

[brb33547-bib-0007] Hirata, K. , Ueda, K. , Komori, M. , Zagar, A. J. , Selzler, K. J. , Nelson, A. M. , Han, Y. , Jaffe, D. H. , Matsumori, Y. , & Takeshima, T. (2021). Comprehensive population‐based survey of migraine in Japan: Results of the ObserVational survey of the Epidemiology, tReatment, and Care Of MigrainE (OVERCOME [Japan]) study. Current Medical Research and Opinion, 37(11), 1945–1955. 10.1080/03007995.2021.1971179 34429000

[brb33547-bib-0008] Iigaya, M. , Sakai, F. , Kolodner, K. B. , Lipton, R. B. , & Stewart, W. F. (2003). Reliability and validity of the Japanese Migraine Disability Assessment (MIDAS) Questionnaire. Headache, 43(4), 343–352. 10.1046/j.1526-4610.2003.03069.x 12656705

[brb33547-bib-0009] Lipton, R. B. , Bigal, M. E. , Kolodner, K. , Stewart, W. F. , Liberman, J. N. , & Steiner, T. J. (2003). The family impact of migraine: Population‐based studies in the USA and UK. Cephalalgia, 23(6), 429–440. 10.1046/j.1468-2982.2003.00543.x 12807522

[brb33547-bib-0010] Lipton, R. B. , Nicholson, R. A. , Reed, M. L. , Araujo, A. B. , Jaffe, D. H. , Faries, D. E. , Buse, D. C. , Shapiro, R. E. , Ashina, S. , Cambron‐Mellott, M. J. , Rowland, J. C. , & Pearlman, E. M. (2022). Diagnosis, consultation, treatment, and impact of migraine in the US: Results of the OVERCOME (US) study. Headache, 62(2), 122–140. 10.1111/head.14259 35076091 PMC9305407

[brb33547-bib-0011] Lipton, R. B. , Stewart, W. F. , Diamond, S. , Diamond, M. L. , & Reed, M. (2001). Prevalence and burden of migraine in the United States: Data from the American Migraine Study II. Headache, 41(7), 646–657. 10.1046/j.1526-4610.2001.041007646.x 11554952

[brb33547-bib-0012] Martelletti, P. , Leonardi, M. , Ashina, M. , Burstein, R. , Cho, S.‐J. , Charway‐Felli, A. , Dodick, D. W. , Gil‐Gouveia, R. , Grazzi, L. , Lampl, C. , Maassenvandenbrink, A. , Minen, M. T. , Mitsikostas, D. D. , Olesen, J. , Owolabi, M. O. , Reuter, U. , Ruiz de La Torre, E. , Sacco, S. , Schwedt, T. J. , … Raggi, A. (2023). Rethinking headache as a global public health case model for reaching the SDG 3 HEALTH by 2030. Journal of Headache and Pain, 24, 140. 10.1186/s10194-023-01666-2 37884869 PMC10604921

[brb33547-bib-0013] Matsumori, Y. , Ueda, K. , Komori, M. , Zagar, A. J. , Kim, Y. , Jaffe, D. H. , Takeshima, T. , & Hirata, K. (2022). Burden of migraine in Japan: Results of the ObserVational Survey of the Epidemiology, tReatment, and Care Of MigrainE (OVERCOME [Japan]) study. Neurology and Therapy, 11(1), 205–222. 10.1007/s40120-021-00305-9 34862581 PMC8857353

[brb33547-bib-0014] Parikh, S. K. , & Young, W. B. (2019). Migraine: Stigma in society. Current Pain and Headache Reports, 23(1), Article 8. 10.1007/s11916-019-0743-7 30739216

[brb33547-bib-0015] Perugino, F. , de Angelis, V. , Pompili, M. , & Martelletti, P. (2022). Stigma and chronic pain. Pain and Therapy, 11(4), 1085–1094. 10.1007/s40122-022-00418-5 35930220 PMC9633893

[brb33547-bib-0016] Rosignoli, C. , Ornello, R. , Onofri, A. , Caponnetto, V. , Grazzi, L. , Raggi, A. , Leonardi, M. , & Sacco, S. (2022). Applying a biopsychosocial model to migraine: Rationale and clinical implications. Journal of Headache and Pain, 23, 100. 10.1186/s10194-022-01471-3 35953769 PMC9367111

[brb33547-bib-0017] Safiri, S. , Pourfathi, H. , Eagan, A. , Mansournia, M. A. , Khodayari, M. T. , Sullman, M. J. M. , Kaufman, J. , Collins, G. , Dai, H. , Bragazzi, N. L. , & Kolahi, A.‐A. (2022). Global, regional, and national burden of migraine in 204 countries and territories, 1990 to 2019. Pain, 163(2), e293–e309. 10.1097/j.pain.0000000000002275 34001771

[brb33547-bib-0018] Sakai, F. , Hirata, K. , Igarashi, H. , Takeshima, T. , Nakayama, T. , Sano, H. , Kondo, H. , Shibasaki, Y. , & Koga, N. (2022). A study to investigate the prevalence of headache disorders and migraine among people registered in a health insurance association in Japan. Journal of Headache and Pain, 23(1), Article 70. 10.1186/s10194-022-01439-3 35733104 PMC9219245

[brb33547-bib-0019] Seng, E. K. , Shapiro, R. E. , Buse, D. C. , Robbins, M. S. , Lipton, R. B. , & Parker, A. (2022). The unique role of stigma in migraine‐related disability and quality of life. Headache, 62(10), 1354–1364. 10.1111/head.14401 36321956

[brb33547-bib-0020] Shapiro, R. , Nicholson, R. , Zagar, A. J. , Kim, Y. , Reed, M. , Buse, D. , Muenzel, J. , Ashina, S. , Pearlman, E. , & Lipton, R. (2022). Identifying factors pertaining to migraine‐related stigma from a novel question set: Results of the OVERCOME (US) study (S4.001). Neurology, 98, (18 Supplement), 2997. 10.1212/WNL.98.18_supplement.2997

[brb33547-bib-0021] Shapiro, R. E. (2020). What will it take to move the needle for headache disorders? An advocacy perspective. Headache, 60(9), 2059–2077. 10.1111/head.13913 32813900

[brb33547-bib-0022] Shapiro, R. E. , Araujo, A. B. , Nicholson, R. A. , Reed, M. L. , Buse, D. C. , Ashina, S. , & Lipton, R. B. (2019). Stigmatizing attitudes about migraine by people without migraine: Results of the OVERCOME study [Conference presentation abstract]. Sixty‐first Annual Scientific Meeting American Headache Society, Philadelphia, PA, United States. Headache, 59(S1), 1–208. 10.1111/head.13549

[brb33547-bib-0023] Shapiro, R. E. , Nicholson, R. A. , Seng, E. K. , Buse, D. C. , Reed, M. L. , Zagar, A. J. , Ashina, S. , Muenzel, E. J. , Hutchinson, S. , Pearlman, E. M. , & Lipton, R. B. (2024). Migraine‐related stigma and its relationship to disability, interictal burden, and quality of life: Results of the OVERCOME (US) study. Neurology, 102(3), e208074. 10.1212/WNL.0000000000208074 38232340 PMC11097761

[brb33547-bib-0024] Stewart, W. F. (1992). Prevalence of migraine headache in the United States: Relation to age, income, race, and other sociodemographic factors. JAMA, 267(1), 64–69. 10.1001/jama.1992.03480010072027 1727198

[brb33547-bib-0025] Stewart, W. F. , Lipton, R. B. , Dowson, A. J. , & Sawyer, J. (2001). Development and testing of the migraine disability assessment (MIDAS) questionnaire to assess headache‐related disability. Neurology, 56(Suppl 1), S20–S28. 10.1212/wnl.56.suppl_1.s20 11294956

[brb33547-bib-0026] Stewart, W. F. , Lipton, R. B. , Kolodner, K. , Liberman, J. , & Sawyer, J. (1999). Reliability of the migraine disability assessment score in a population‐based sample of headache sufferers. Cephalalgia, 19(2), 107–114. 10.1046/j.1468-2982.1999.019002107.x 10214536

[brb33547-bib-0027] Takeshima, T. , Ueda, K. , Komori, M. , Zagar, A. J. , Kim, Y. , Jaffe, D. H. , Matsumori, Y. , & Hirata, K. (2022). Potential unmet needs in acute treatment of migraine in Japan: Results of the OVERCOME (Japan) study. Advances in Therapy, 39(11), 5176–5190. 10.1007/s12325-022-02289-w 36089637 PMC9525323

[brb33547-bib-0028] Vaghi, G. , de Icco, R. , Tassorelli, C. , Goadsby, P. J. , Vicente‐Herrero, T. , & de La Torre, E. R. (2023). Who *cares* about migraine? Pathways and hurdles in the European region—Access to care III. Journal of Headache and Pain, 24, 120. 10.1186/s10194-023-01652-8 37653377 PMC10472594

[brb33547-bib-0029] Woldeamanuel, Y. W. , & Cowan, R. P. (2017). Migraine affects 1 in 10 people worldwide featuring recent rise: A systematic review and meta‐analysis of community‐based studies involving 6 million participants. Journal of the Neurological Sciences, 372, 307–315. 10.1016/j.jns.2016.11.071 28017235

[brb33547-bib-0030] Young, W. B. , Park, J. E. , Tian, I. X. , & Kempner, J. (2013). The stigma of migraine. PLoS One, 8(1), e54074. 10.1371/journal.pone.0054074 23342079 PMC3546922

