# Supplementary material for: Underrecognition of migraine‐related stigmatizing attitudes and social burden: Results of the OVERCOME Japan study
Source: Brain Behav. 2024 Jul 25;14(7):e3547. doi: 10.1002/brb3.3547 (PMC11272416; doi:10.1002/brb3.3547)
Supplement: Supplementary file 1 — TABLE S1 Questions about stigmatizing attitudes toward migraine in the 11‐item attitudinal questionnaire. FIGURE S1 Proportion of respondents without migraine (non‐migraine group) who answered “sometimes,” “often,” or “very often” to the attitudinal questions about migraine by their relationship to the person with migraine [file BRB3-14-e3547-s001.pdf]

SUPPLEMENTARY MATERIAL

Stigmatizing attitudes and social burden about migraine: Results of the OVERCOME JAPAN study

Hisaka Igarashi[1], Mika Komori[2], Kaname Ueda[2], Anthony J. Zagar[3], Dena H. Jaffe[4], Yasuhiko Matsumori[5], Takao Takeshima[6], Koichi Hirata[7]

AUTHORS' AFFILIATIONS

[1]Fujitsu Clinic, Kawasaki, Japan

[2]Eli Lilly Japan K.K., Kobe, Japan

[3]Eli Lilly and Company, Indianapolis, Indiana, USA

[4]Cerner Enviza, an Oracle Company (formerly Kantar Health), Jerusalem, Israel

[5]Sendai Headache and Neurology Clinic, Sendai, Japan

[6]Tominaga Hospital, Osaka, Japan

[7]Dokkyo Medical University, Mibu, Japan

CORRESPONDING AUTHOR

Mika Komori

Japan Drug Development and Medical Affairs

Eli Lilly Japan K.K.

5-1-28, Isogamidori, Chuo-ku

Kobe-shi 651-0086

Japan

Tel: +81-78-242-8391

Email: komori_mika@lilly.com

LIST OF TABLES AND FIGURES

TABLE S1 Questions about stigmatizing attitudes toward migraine in the 11-item attitudinal questionnaire.

FIGURE S1 Proportion of respondents without migraine (non-migraine group) who answered "sometimes," "often," or "very often" to the attitudinal questions about migraine by their relationship to the person with migraine.

TABLE S1 Questions about stigmatizing attitudes toward migraine in the 11-item attitudinal questionnaire

**Question items: "How often have you felt that people with migraine…"**
1. Use their migraine or severe headache as a way to get out of work or school commitments?
2. Use their migraine or severe headache as a way to get out of family or social commitments?
3. Use their migraine or severe headache as a way to get attention?
4. Use their migraine or severe headache as a way to get pain medications they do not really need?
5. Have migraine or severe headache as a result of their own unhealthy behavior(s)?
6. Should not bother seeing a doctor about migraine or severe headache?
7. Should be able to easily treat their migraine or severe headache?
8. Exaggerate the symptoms associated with migraine or severe headache?
9. Exaggerate the burden of the migraine or severe headache?
10. Try to hide their migraine or severe headache from others?
11. Make things difficult for their coworkers or supervisor?

The response for each question was scored on a five-point Likert scale, with responses of "never," "rarely," "sometimes," "often," and "very often," or "don't know."

FIGURE S1 Proportion of respondents without migraine (non-migraine group) who answered “sometimes,” “often,” or “very often” to the attitudinal questions about migraine by their relationship to the person with migraine. The answers were scored on a five-point Likert scale, with responses of “never,” “rarely,” “sometimes,” “often,” and “very often,” or “don’t know.”

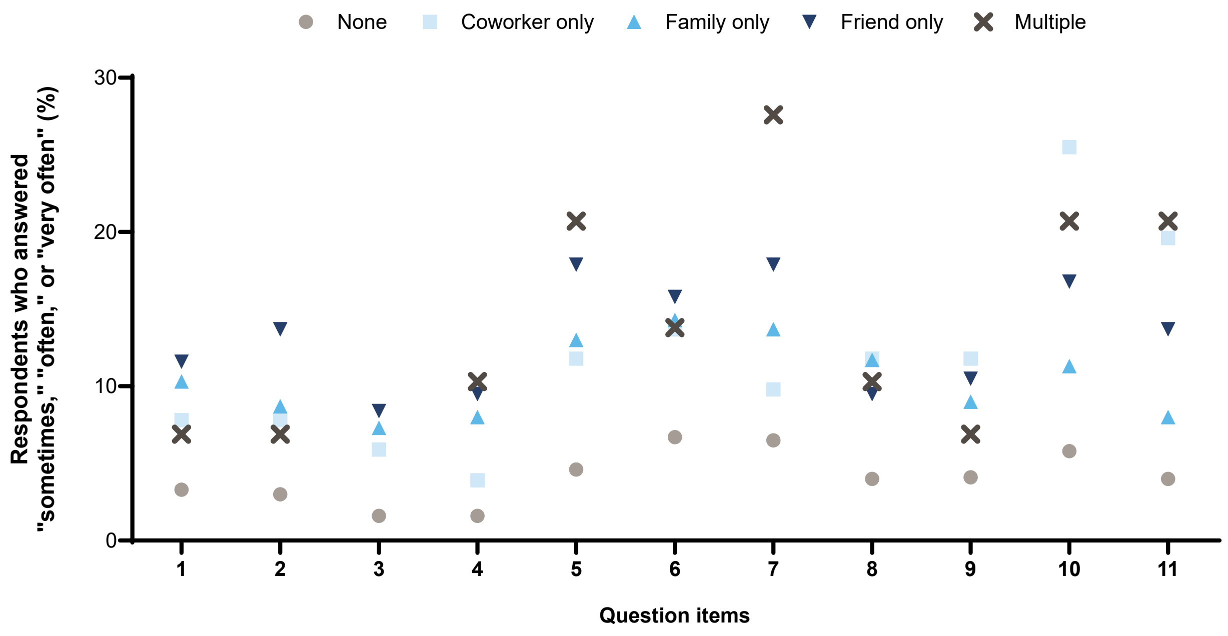

1. Use their migraine or severe headache as a way to get out of work or school commitments?
2. Use their migraine or severe headache as a way to get out of family or social commitments?
3. Use their migraine or severe headache as a way to get attention?
4. Use their migraine or severe headache as a way to get pain medications they do not really need?
5. Have migraine or severe headache as a result of their own unhealthy behavior(s)?
6. Should not bother seeing a doctor about migraine or severe headache?
7. Should be able to easily treat their migraine or severe headache?
8. Exaggerate the symptoms associated with migraine or severe headache?
9. Exaggerate the burden of the migraine or severe headache?
10. Try to hide their migraine or severe headache from others?
11. Make things difficult for their coworkers or supervisor?
